# Periorbital injections of botulinum toxin a: a novel therapeutic option for convergence spasm in neuropsychiatric disorders

**DOI:** 10.1007/s00415-021-10613-7

**Published:** 2021-05-28

**Authors:** Kristina Hess, Moritz Schmitt, Bettina Wabbels

**Affiliations:** Department of Ophthalmology, University Hospital Bonn, University of Bonn, Ernst-Abbe-Str. 2, 53127 Bonn, Germany

**Keywords:** Convergence spasm, Spasm of the near reflex, Botulinum toxin, BoNTA, Functional neurological disorders

## Abstract

**Purpose:**

Convergence spasm (CS, spasm of near reflex) is characterized by transient attacks of convergence, miosis and accommodation, often associated with functional neurological disorders. To date, no simple and efficient treatment option is available for CS. This study investigates whether periorbital botulinum toxin injections as used in essential blepharospasm are also a treatment option in these patients.

**Methods:**

All patients with convergence spasm having been treated with periorbital BoNTA injections in the department of neuro-ophthalmology were identified. Data were extracted from patient files concerning details and subjective effectiveness of botulinum toxin injections and relation to psychiatric or neurological disorders. Patients reporting with a history of closed-head trauma or organic neurologic pathologies possibly causing CS were excluded. A telephone assessment with a standardized questionnaire was performed to evaluate mental health issues as a trigger, as well as the long-term effect and satisfaction with periorbital injections.

**Results:**

Of 16 patients treated with periorbital botulinum toxin injections for convergence spasm, 9 patients reported depression and/or anxiety disorders ongoing or in the past. A median number of 3 injections (range 1–13) was administered with a variable effect (relief of symptoms) between no effect and effect of up to more than 12 weeks. A longitudinal follow-up revealed ongoing symptoms in five patients.

**Conclusions:**

Periorbital botulinum toxin injections are less invasive than injections in the medial rectus muscle and can be a bridging therapeutic option in patients with CS. Mental health exploration is important due to psychiatric comorbidity.

## Introduction

Convergence spasm (also known as spasm of near reflex) is characterized by a transient and intermittent appearance of bilateral convergence, miosis and accommodation mostly symptomatic to patients as blurred or double vision [1–3]. First described by von Graefe in 1856, a plethora of neurological and psychiatric diseases have been identified as underlying causes to date, among others including encephalitis, metabolic encephalopathy, tabes dorsalis, post-myelography, vertebrobasilar ischemia, brain stem pathology and multiple sclerosis [4–6].

The clinical signs of patients with convergence spasm are often misinterpreted as sixth nerve palsy, the main differential diagnosis, and patients are then subjected to further diagnostic tests or even invasive procedures such as lumbar puncture [4].

However, convergence spasm can often be a presenting symptom of functional neurological disorders. This in turn can be comorbid with stress, anxiety and personality disorders or such features may be absent [7, 8].

Previous publications, therefore, differentiate non-organic from organic convergence spasms, the latter due to organic neurologic pathologies (e.g., after closed-head traumata, or neurological diseases as mentioned above), since the course of the symptoms and treatment options are different [2, 8–13].

Several treatment options for both types of convergence spasm have been proposed including cycloplegic eye drops (with resulting blurred vision in the near distance and the need for additional plus lenses), prisms or squint surgery, most of them with limited effect [14–17]. Some patients benefit from repeated intramuscular Botulinum toxin A (BoNTA), injections in one or both M. rectus medialis, as shown in data of 15 patients of our department published in 2002 [18]. Seven of 15 patients reported no long-term effect of intramuscular BoNTA.

However, there are several disadvantages of intramuscular injections in the medial rectus muscle, such as the requirement of special expertise in EMG recording of extraocular eye muscles. In addition, intramuscular injections are not recommended in patients under anticoagulant therapy due to the risk of peri- and retroorbital bleedings.

**Fig. 1:**
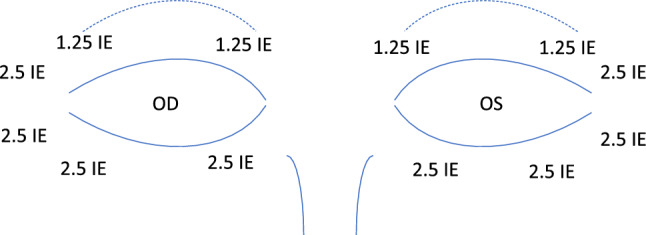
BoNTA scheme used for initial treatment in convergence spasm patients in the University Eye Hospital Bonn similar to the standard treatment in essential blepharospasm. A dilution of 4 ml sodium chloride (NaCl) per 100 IE BoNTA (ona- or incobotulinumtoxin) was used

In our department, periorbital injections of BoNTA of the periorbital regions (M. orbicularis oculi, see Fig. 1) are regularly performed in patients with essential blepharospasm in a dedicated clinic.

Due to the disturbing side effects of cycloplegic eye drops in patients with convergence spasm and the common comorbidity of psychogenic disorders of these two entities, we initiated a periorbital BoNTA therapy in a patient, that could not discontinue his oral anticoagulation. After reporting an unanticipated success in this patient, all patients with convergence spasm were treated with bilateral periorbital injections of BoNTA as first-line therapy since then. Injections in the M. rectus medialis were only performed in patients where the periorbital injections were not successful.

Periorbital injections are generally performed without EMG recording and also in patients under anticoagulation, similar to patients with essential blepharospasm. An initial dose of 12.5–15.0 IE ona- or incobotulinum toxin (Botox^®^, Xeomin^®^) per eye was usually administered including pretarsal injections, the dosage could be adapted according to patient needs (Fig. 1).

The aim of this study was to evaluate the potential of periorbital BoNTA injections in patients with convergence spasm as a novel treatment option.

## Methods

All files of patients treated with periorbital BoNTA injections in the M. orbicularis oculi (“periorbital injection”) for convergence spasm between 2008 and 2019 were analyzed in this study. All patients presented at the clinic of Neuro-ophthalmology and Orthoptics at the Department of Ophthalmology, University of Bonn, which is a tertiary care referral center for botulinum toxin injections with a dedicated weekly BoNTA clinic.

Inclusion criteria were a diagnosis of convergence spasm based on orthoptic and ophthalmological examination followed by at least one periorbital injection of BoNTA.

The patients’ files were evaluated retrospectively for referral diagnosis, ophthalmological and systemic diseases and specific psychiatric or neurological disorders at presentation and during the course of visits.

Exclusion criteria were the history of a closed-head trauma or organic neurological diseases possibly causing convergence spam (e.g., several patients with intracranial hemorrhages).

Furthermore, details of BoNTA injections and treatment effects were assessed including subjective change of symptoms, duration of relief and complications. The treatment effect reported by the patients was categorized into three groups: long (12 weeks or longer), intermediate (7–11 weeks) or short-term duration of symptom relief (6 weeks or less).

After a positive vote from the institutional ethics committee (Appr. Nr. 139/19, University of Bonn, Bonn, Germany), a letter of notification regarding our study was sent to all patients in March 2020 with the possibility to decline participation.

All patients were then contacted by a standardized telephone interview for an assessment of their long-term effects of the BoNTA treatments and their current health condition.

Statistical analysis was performed using GraphPad Prism version 8.0 (GraphPad Software, San Diego, CA, USA).

## Results

### Patient characteristics

A total of 16 patients were identified and included in this study (Table 1). The median age at first presentation was 40 years (IQR 26; 44.5, range 11–52 years], 9 female).

**Table 1:** Patient characteristics of all patients with convergence spasm treated with periorbital BoNTA

ID	Age at BL (years)	Gender	Number of periorbital injections	Median release of symptoms after periorbital injections (weeks)	Referral due to CS?	Previous therapeutic approaches	Ophthalmological history and findings	Additional treatment	Neurological history/evaluation and MRI	Psychiatric diseases in medical history
1	23	M	4	8	No	None		Antidepressant medication, relaxation exercises	wnl	ADHD, depression
2	41	F	1	0	No	Strabismus surgery	High hyperopia			Mental handicap since birth
3	15	F	3	6	No	Cycloplegia	Four squint surgeries in the past	Therapy of anorexia nervosa, antidepressant		Anorexia, borderline syndrome
4	42	F	1	8	Yes	Cycloplegia, prisms			wnl	Depression
5	49	M	6	0	No	None	Esotropia, hyperopia	Relaxation exercises		Anxiety disorder, depression
6	40	M	3	12	No	None	Unilateral high hyperopia, tried glasses and contact lenses	Relaxation exercises, correction of hyperopia	wnl	
7	52	F	12	12	No	Prisms		Relaxation exercises		
8	40	M	5	> 12	No	None			wnl, headaches attributed to overuse of analgetics	
9	20	F	4	12	No	None	Esophoria and hyperopia	Relaxation exercises		
10	11	M	1	3	No	None				In psychological treatment
11	28	M	13	3	Yes	None	Squint surgery in the past	Antidepressant medication, inpatient in psychosomatic clinic	wnl	In psychological treatment
12	44	F	3	2	No	None	Orbital floor fracture 8 years before, squint surgery 3 years before			Depression
13	27	F	2	5	No	Prisms, occlusion contact lens, 2 strabismus surgeries	Myopia	Psychologic counseling, changed job	wnl	
14	52	F	1	1	No	None	Intermittent exotropia, myopia	Coaching, patient preferred occlusion CL	wnl	
15	46	M	2	10	No	Prisms	Esotropia, hyperopia	Relaxation exercises, squint surgery		
16	35	F	1	12	No	Cycloplegia			wnl	

The first ten patients were additionally assessed longitudinally in the telephone interview (details see Table 2)

*ID* identification number, *BL* baseline, *po* periorbital, *MR* medial rectus muscle, *CS* convergence spasm, *ADHD* attention-deficit hyperactivity disorder, *wnl* within normal limits

Two patients were referred due to the diagnosis of convergence spasm, all other patients (*n* = 14) were referred for either general consultation in our Neuro-ophthalmology clinic or with another diagnosis, e.g., sixth nerve palsy or esotropia.

Previous therapeutic approaches without sufficient effect included prisms (*n* = 3), cycloplegic eye drops (*n* = 3) and squint surgery (*n* = 2). Nine patients had not received specific treatment before referral. Duration of symptoms until presentation was between 6 months and 4 years.

Eight of 16 patients presented at baseline with a psychiatric diagnosis in their history or were in current psychological and/or medical treatment with depression (*n* = 4) and anxiety disorder (*n* = 1) as the predominant comorbidity. One patient had a mental handicap since birth and two patients were in psychological treatment awaiting a confirmed diagnosis. Eight patients had no history of psychiatric disorders, but three of them indicated extreme mental stress situations when the eye problems started, two patients at work and one in private life (ID 9, 13, 14 in Table 1).

All patients had full orthoptic and ophthalmologic examination at every visit and all refractive errors were fully corrected, except in one patient, where full correction of a unilateral hyperopia was only accepted after two injections of BoNTA (ID 6 in Table 1). In all patients, convergence spasm could be documented initially including miosis and overaccomodation.

Three patients had a history of squint surgery for convergent strabismus in childhood, not related to the actual problems. Two further patients had esotropia, and one patient had an intermittent exotropia.

The most common additional ophthalmological symptom alongside the convergence spasm could be attributed to a dry eye syndrome (*n* = 10, 62.5%) and was in all cases confirmed in our examination.

### BoNTA injections

All 16 patients received periorbital injections with a median number of 3 injections (range 1–13). Figure 1 shows the injections points and dosage of BoNTA for initial treatments. In patients with more than one injection, dosage was modified according to side effects and patients’ needs: 13 patients were treated with 12.5 IE per side, in two patients dose was reduced to 7.5 IE/10.0 IE, in one patient dosage was 17.5 IE.

Five patients were treated with only one injection, two of these did not want to continue the treatment, one patient reported long travel times as the reason (ID10), in the other patients, symptoms resolved after one treatment (Table 1).

Periorbital BoNTA injections induced a subjective relief of symptoms for a median of 5.5 weeks, with high interindividual variability (0–12 weeks) (Table 1).

Five patients had immediate and long-lasting resolution of symptoms (12 weeks or longer), three patients showed benefit over an intermediate period and five had benefit, but the treatment effects lasted less than 6 weeks and three had no benefit (maximum: 1 week). Two of the patients with no benefit did not want to receive a second injection.

In those patients receiving more than one injection, injection intervals were 3 months, or more when patients preferred longer intervals. Three patients (ID 1, 3, 8) even had intervals up to 1 year. One patient (ID 9) initially had two injections before his symptoms resolved. This patient came back for another injection 2 years later and for a fourth injection 3 more years later. No patient asked for or had shorter injection intervals than 3 months.

As a BoNTA-associated complication, one patient reported a mild hemifacial weakness and one patient reported a mild and transient ptosis.

Five patients had completed a symptom diary with daily requests to state the effect of the BoNTA treatment using a 0–100% scale of symptom relief. Four patients exhibited an increasing effect in the first weeks with high interindividual variability regarding the subjective effect size of 15–100% and duration of the effect (7 weeks to > 12 weeks).

Of the three patients that did not benefit from the periorbital treatment, one patient (ID 2 in Tables 1 and 2) that reported no effect at all over the time period of 12 weeks suffered from a mental handicap since birth, therefore, possibly being an atypical convergence spasm patient.

**Table 2:** Patient characteristics of the telephone assessment

ID	Follow-up time (years)	Diplopia provocable	Trigger	First episode due to stress situation?	Current impact on Life Quality (scale 1–10)	Satisfaction with effect (scale 1–10)	Satisfaction with duration (scale 1–10)	Recommendation for other patients as a treatment option? (scale 1–10)	BoNTA or alternative treatment option superior?
1	3.8	No		Yes, stress	5	6	3	6	No alternative therapy
2	10.3	Yes	Stress	N/A	3	5	4	5	No alternative therapy
3	10.6	No		Yes, domestic violence	0	10	10	8	BoNTA superior
4	10.3	No		Yes, stress	0	5	5	6	BoNTA superior
5	8.9	Yes	Fine motor skills, also daily form	Yes	6	6	3	7	BoNTA superior
6	3.5	No		No	0	7	5	7	No alternative therapy
7	13.7	Yes	Stress, tiredness	Yes, stress	5	9	5	9	BoNTA superior
8	1.6	Yes	Stress, tiredness	no	7	10	5	10	No alternative therapy
9	3.5	No		Yes, exam	5	10	4	8	BoNTA superior
10	8.5	Yes	Stress	No	5	9	2	3	Lamotrigine currently superior

*BoNTA* botulinum toxin A

ID numbers refer to the IDs in Table 1. The follow-up period is the time between the first visit in our department and the telephone assessment

Another patient (ID 5 in Tables 1 and 2) received intramuscular injections in the medial rectus muscle after initial periorbital treatment. The switch to MR injections occurred after six periorbital injections and was taken into account due to insufficient relief of symptoms after the last periorbital BoNTA injection. The third patient (ID14) preferred occlusion contact lens although she did not note any side effects.

Clinical follow-up times after the first BoNTA injection ranged from 3 months to 5 years (mean 2.9 years, SD 1.8 years).

### Long-term follow-up assessment (telephone interview)

Ten of 16 patients could be contacted for an evaluation of their past BoNTA treatment (Table 2). The time since the last treatment was 6.5 ± 3.2 years (mean ± SD, range 6–139 months). None of the patients declined to be contacted, however, six patients could not be contacted due to relocation or change of telephone numbers.

Five of ten patients reported that convergence spasm was still provocable in specific situations. Of these, four patients named stress and strain as a trigger.

Although only five patients are without any symptoms in all situations, the contentment regarding periorbital BoNTA injections among all patients was high: with a median of 8 (range 5–10), patients were happy with the treatment effect. However, the satisfaction regarding the duration of relief was lower with a median of 4.5 (range 2–10).

## Discussion

Convergence spasm (spasm of the near reflex) is a complex disorder with unclear etiology. There are no guidelines for therapy yet, while different approaches have been proposed.

In convergence spasm related to functional neurological disease, an associated psychiatric comorbidity may be present but is not always found. Interventions and therapy of the underlying disorders can lead to relief and discontinuation of symptoms [8, 12, 19, 20]. Monocular occlusion has been proposed for symptomatic treatment of double vision but leads to reduced stereopsis and impairs the appearance of patients [21, 22]. Orthoptic exercises, prescription of near vision glasses and prism glasses, as well as squint surgery have been reported with only limited effect [14, 16]. Pharmacological approaches are not easy since the molecular target is unclear. Cycloplegic eye drops are sometimes used but lead to impaired near vision with the need of additional plus glasses and to problems with glare in the mostly young patients.

This is the first study to describe periorbital BoNTA injections as a treatment option for convergence spasm. Periorbital injections might be advantageous in patients with convergence spasm often associated with comorbidities such as depression, anxiety or post-traumatic stress disorders or occurring in periods of stressful life events to bridge the time until additional interventions as medication, psychologic counseling or stress-reducing strategies are effective [7, 8].

Compared to medial rectus muscle injections, periorbital injections are less invasive, less painful, overcorrection resulting in double vision is unlikely and the approach is more widely and easier available since no electromyographic surveillance is necessary. In addition, no discontinuation of anticoagulants is necessary.

There is little information regarding the long-term natural history of convergence spasm published to date. The 10 patients with spasm of the near reflex published by Rutstein et al. [23] mostly had an onset of symptoms within a year, also three patients have complaining of problems for 2/4 and 14 years, respectively. Mean follow-up was 9.6 months (up to 30 months). Our patients were suffering from the disease between 6 months and 4 years prior to their first injection and had clinical follow-ups between 3 months and 5 years. In the telephone interview, five of the ten patients still had the symptoms on provocation after a mean of 8.6 ± 5.1 years (range 1.6–13.7 years) since the first visit at our department, showing that convergence spasm can be a long-lasting problem.

Another interesting finding is the fact, that five patients had esotropia, three of these had surgery in the past, not related to the actual problems. One patient had intermittent exotropia. This may indicate that an underlying strabismus may make patients more prone to develop convergence spasm. In the study of Rutstein [23] describing 17 patients with accommodative spasms, ten of these having convergence spasm, only one patient had strabismus.

The effect of periorbital BoNTA injections in our study is to date not easily explainable, but one hypothesis includes the lower eyelid pressure: Namiguchi et al. have shown a significantly lower eyelid pressure in patients with blepharospasm after BoNTA injections [24]. Another investigation has shown significantly higher eyelid pressure values in patients with dry eye syndrome, which was the most common ophthalmological finding in our cohort [25]. The reduced pressure of the eyelids to the surface can lead to a general relaxation of the periorbital area, and therefore, a possible relaxation also of the extraocular muscles. This may be similar to the previously reported effect of the facial feedback known in patients with depression injected with botulinum toxin [26, 27]. However, further analysis regarding the effect of BoNTA injections in general and specifically for these diseases is warranted.

Another explanation that has to be discussed is a sole or main placebo effect. A randomized, double-blind, placebo-controlled study using botulinum neurotoxin in patients with functional movement disorders of other muscle groups has shown no evidence of improved outcome in the treatment group compared to placebo, but potential for improvement of symptoms due to placebo effects affecting both groups [28].

Especially due to the associated comorbidities and possible underlying stress situations as trigger, a “novel” and invasive treatment may have contributed to a positive perception of the intervention in our study. Therefore, further studies comparing this approach in comparison to other interventions are needed. Vice versa it can be concluded that if a high placebo effect is suspected, more invasive treatments, such as intramuscular injections should be initiated cautiously in patients with underlying psychiatric diseases and further studies on placebo therapies are warranted.

An additional risk of this treatment is the recurrence with the possibility of provoking a dependency on the injections and prolongation of the underlying disease course, although we could not see this in our patients. This underscores the importance of detailed consenting and explanation regarding the role as a supporting therapy, as done in our clinic. In contrast, we had several patients with good effect who told us that they would actually not need further treatment. In these cases, we offered to come back whenever they would need it and four patients came back for single injections after one to 3 years. Unlike in patients with blepharospasm, where injection intervals can be reduced to 6 weeks as needed, we would not recommend to do so in patients with convergence spasm.

From our clinical experience, no or only little effect of periorbital BoNTA injections can be expected in patients with a confirmed neurological disease, especially in patients after intracranial hemorrhage. These patients seem to profit from antiepileptic medication or from frosted glasses to prevent double vision in the event of convergence spasm attacks. However, the existence of organic convergence spasm is controversial, since the event of a trauma or accident can also serve as a trigger for functional symptoms, as shown for other neurological diseases [29].

Due to the unknown etiology, physicians should keep in mind that the assessment of neurological and psychiatric diseases as well as psychological counseling if needed tackles the underlying trigger and is, therefore, crucial for the long-term well-being of the patient. In our study, the patients’ history was taken in an ophthalmology department, and therefore, possibly not specifically focused on underlying psychiatric or psychosomatic comorbidities, but of the eight patients which were not already in neurological or psychiatric treatment, five had a neurological workup. A precise record of the patients’ current life situation, possibly family history and coping strategies nevertheless may reveal further and in some cases treatable coherences for which convergence spasm is only a secondary finding.

This study had limitations. First, the small sample size is a common problem in research on rare diseases. In addition, the retrospective nature of this study warrants further research with a double-masked sham-control approach, e.g., a control group with injection of NaCl would be desirable to confirm that the effect can be ascribed to BoNTA. However, the obvious and distinct effect of BoNTA on eyelid and other muscles rules out some study designs which would be desirable due to high interindividual variability and low sample sizes. In addition, studies to further analyze the co-existence of psychiatric disorders are necessary to give insights to the disease etiology.

Finally, it cannot be distinguished, to what extend these patients’ symptoms would have resolved without any treatment or with sole treatment of the underlying comorbidities (if applicable). Nevertheless, we could show for the first time that convergence spasm may be a long-lasting problem for patients.

## Conclusions

Periorbital BoNTA injections provide a less invasive therapeutic option than injections in the medial rectus muscle for patients with convergence spasm and it could be helpful to have an easy to perform treatment option in these patients often suffering from double vision and blurred vision for many years. Periorbital BoNTA injections can specifically be an option in patients with psychiatric or psychosomatic comorbidities or for patients with stressful life events which often have already high levels of life quality restriction due to their disease. Since at least a part of the effect might be attributed to placebo, further placebo-controlled studies are needed.

## Data Availability

KH and BW had full access to all the data in the study and take responsibility for the integrity of the data and the accuracy of the data analysis. KH and BW conducted and are responsible for the data analysis. Original data will be shared from the corresponding author on reasonable request.
